# A perspective on the sharing of docking data

**DOI:** 10.1016/j.dib.2023.109386

**Published:** 2023-07-07

**Authors:** Samia Aci-Sèche, Stéphane Bourg, Pascal Bonnet, Joseph Rebehmed, Alexandre G. de Brevern, Julien Diharce

**Affiliations:** a Institut de Chimie Organique et Analytique (ICOA), UMR CNRS-Université d'Orléans 7311, Université d'Orléans BP 6759, Orléans Cedex 2, 45067, France; b Department of Computer Science and Mathematics, Lebanese, American University, Beirut, Lebanon; c Université Paris Cité and Université des Antilles and Université de la Réunion, INSERM, Biologie Intégrée du Globule Rouge, UMR_S 1134, DSIMB Bioinformatics team, 75014 Paris, France

**Keywords:** 3D coordinates, Docking, Files, SDF, Sharing, FAIR principles

## Abstract

Computational approaches are nowadays largely applied in drug discovery projects. Among these, molecular docking is the most used for hit identification against a drug target protein. However, many scientists in the field shed light on the lack of availability and reproducibility of the data obtained from such studies to the whole community. Consequently, sustaining and developing the efforts toward a large and fully transparent sharing of those data could be beneficial for all researchers in drug discovery. The purpose of this article is first to propose guidelines and recommendations on the appropriate way to conduct virtual screening experiments and second to depict the current state of sharing molecular docking data. In conclusion, we have explored and proposed several prospects to enhance data sharing from docking experiment that could be developed in the foreseeable future.

## 1 Background

Drug discovery is nowadays one of the most prevalent fields in scientific research. Hence, the number of drugs proposed by the Food and Drug Administration (FDA) was 50 in 2021, with a 5-year average of 51, whereas it was almost twice less ten years ago [1]. It may be emphasized that the role of computational approaches is directly linked to the great improvement of the research around drug discovery during this last decade. This period has also been marked by an important technical revolution in theoretical calculations and numerical simulations, with the adoption of Graphical Process Unit (GPU) hardware for scientific calculations [2,3]. Previously, GPU were notably known to support intense calculation for 3D modelling or visualization.

By consequence, Computer-Aided Drug Design (CADD) has known a true expansion during the 2010’s. For instance, out of the entire pool of commercially available drugs, 70 compounds have been designed using one (or more) computational approach and among them, half has been developed during this decade [4]. Indeed, computer techniques propose a large panel of possible models, from the molecular to the analytic level. Molecular modeling, using techniques such as homology modeling, molecular docking, or molecular dynamics simulations, is used to investigate biological mechanisms associated to the drug, including binding mode, association/dissociation processes, conformational changes, etc. [5], [6], [7]. In addition, other techniques, such as machine learning methods, are used for the prediction of specific properties such as activities, kinetics or ADME-Tox aspects, building models called Quantitative Structure Activity/Property Relationship, (QSAR/QSPR models) [8,9]. Moreover, over the past decade, we have witnessed the emergence of deep learning approaches, which have been supplied by the growth in computational power and the amount of available data [10]. Very recently, a comprehensive review covering all methodologies in CADD has been published, providing a valuable resource for those seeking more details. [11].

Amongst those different methodologies, Virtual Screening (VS) is one of the most used in CADD to explore molecular databases and find interesting compounds for a considered target. Even it is by essence very expensive and time-consuming, it remains the method of choice for hit identification and optimization. Virtual screening may be divided in two main approaches: the structure-based and the ligand-based approaches. This last one, requiring only the knowledge of molecules binding to the biological target of interest, was often used for molecular similarity, pharmacophore query or QSAR/QSPR modeling. But the most widespread method employed in the drug discovery is the technique named molecular docking [4,12].

It could be of two main natures: small molecule (ligand) to macromolecule or macromolecule to macromolecule, majority represented by ligand-protein and protein-protein docking respectively. While sharing the fundamental theoretical principles, these methodologies are distinct due to the inherent nature of the entities involved and the complexity of their interface [13,14]. In this article, we will only focus on the ligand-receptor paradigm.

**Fig. 1:**
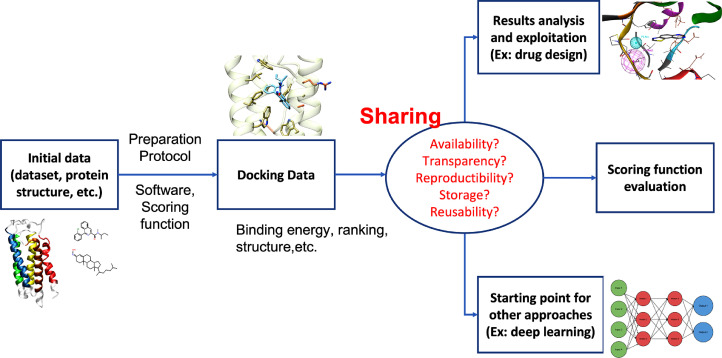
Scheme pointing out the main points to effectively generate and reuse relevant docking data.

As depicted earlier, the importance of molecular docking in drug discovery is substantial and requires no further proof. However, a crucial question is often forgotten: the free availability of docking data to the scientific community. This question is not new and concerns every aspect of the science and every scientist worldwide. It has been put forward in the article of Wilkinson et al. in 2016 [15], by the publication of the FAIR (Findable, Accessible, Interoperable, Reusable) principles. This study is the cornerstone for a new way of thinking in science, leading to the philosophy of sharing as soon as possible data of studies, with sufficient indications, precisions and clarity for a possible reuse. Within such context, the data to be shared extend beyond the outcomes of docking campaigns or to the docking parameters employed. To ensure a proper reuse and reproducibility of these results, it is also necessary to provide the structural data used to set up the docking experiment as well as the detailed protocols employed to prepare the structures or to rank and analyze the docked compounds. Fig. 1 proposes a global view of all the concepts associated with the sharing of the data generated by docking calculations.

The present article aims to provide an overview of the current status of docking data, starting with the good practices for generating meaningful docking results, and then depict the current state of data sharing in this field and explore potential avenues for improvement and future developments.

## 2 Recommendations

### 2.1 Generation of docking data: basis and reflexes

Generating docking results is not as simple as it seems. Firstly, the choice of a correct protein structure and a set of ligands is not trivial. Moreover, those elements need to be carefully and meticulously prepared because they are essential for the relevance of the data that will be generated (see Fig 1).

#### 2.1.1 Preparation of the protein for docking calculations

Protein structures can be solved by experimental methods or predicted by means of computational approaches. The Protein Data Bank (PDB) is an online database ([16], www.pdb.org, accessed on May 5th 2023) composed by more than two hundred thousand of protein structures, solved by experimental means such as X-Ray crystallography, NMR or cryo-EM techniques. Therefore, mining this database is the first thing to do when one is searching for a structure for a docking experiment. But those structures need to be prepared before the docking. First, structures can sometimes have some defects, especially the non-resolution of some protein portions. Generally, those parts correspond to the most mobile parts in the structure, which are often coupled to non-truly important and functional regions, and are not always required for the docking calculation. However, the completion of the protein is often mandatory to reach results with real meaning. Indeed, one must keep in mind that sometimes precautions must be taken when the missing part concerns functional domains. Completion can be done with homology modeling (for example, Modeller [17]), or deep learning approaches which are very numerous now, e.g. AlphaFold [18], CollabFold [19], RoseTTAfold [20]. It is worth noting that these methodologies can propose structure models for protein that have not been experimentally solved. However, care must be exercised regarding the quality of such models, just as with PDB structures.

Second, predicting the protonation state of residues is one of the most important tasks to realize before performing the docking calculation, based on the pH decided by the user. Indeed, charges must be put on every atom of the protein for a good conduction of the docking process. In this matter, some tools are very useful such as the H++ [21] or ProPKA [22,23] servers or standalone programs like MCCE or PyPKA [24,25]. Particular attention and careful consideration should be given to buried residues. This is due to the fact that the local pH within the protein structure can vary significantly from the physiological conditions, as these residues are inaccessible to water molecules and are concealed within the protein fold. Consequently, the pKa values of those amino acids may vary, leading to potentially important consequences on the docking outcomes.

Finally, the structure of the protein deposited in the PDB database is mainly (except RMN structures) one rigid conformation, while proteins are dynamic macromolecules. Alternative stable conformations may exist for a binding site and an even limited change of its conformation can lead to the recognition of new interacting molecules. Thus, considering several conformations of the protein, depending of the nature and the flexibility of the cavity, is necessary in order to obtain more rigorous results with docking methodologies. When other conformations are not available, different methodologies such as molecular dynamics (MD) simulations [3] or normal mode analysis [26] could be employed.

#### 2.1.2 Selection and preparation of the ligand dataset

The next point to consider before the docking calculation itself is the selection and the preparation of the ligand dataset. The number of chemical databases, meaning a library which lists several compounds, is nowadays quite enormous. In 2021, Sabe et al. [4] documented a list of 117 databases that have been consistently used for the last six years, amongst them ZINC [27], ChEMBL [28], DrugBank [29] and PubChem [30]. Some efforts have also been dedicated to the conception of more focused chemical libraries [31], [32], [33], [34]. Those databases could be commercial or public and list lots of information in addition to the name and structure of the molecule of interest. We may also add to this list all the private chemical libraries specific to a particular project.

Hence, many steps of preparation must be realized before the docking because those databases are not prepared for the calculation. The first step aims to filter the entries to remove the erroneous compounds and duplicates and keep only one molecule per entry. Second, a conversion from 1D or 2D format (classical format for online chemical databases) to 3D coordinates must be carried out in order to generate usable compounds for docking calculations. The generation of 3D coordinates is done using 4 different steps: (1) tautomer elucidation, (2) hydrogen atoms addition, (3) definition of the several stereoisomers and (4) generation of stables conformers, with a particular attention to ring conformations. One must also notice that some steps could be avoided if the initial file possesses those information (especially the tautomer and the asymmetry of carbon atoms). Several dedicated programs have been proposed in the literature for this purpose such as VSprep [35] or Gypsum-DL [36].

#### 2.1.3 Docking calculation

Once the preparation steps are completed, the prepared protein target and chemical libraries can be submitted to the docking process. There are many docking software, such as Autodock Vina [37], Glide [38], Gold [39], DOCK [40], rDock [41], PLANTS [42], etc. Numerous published papers and reviews have precisely described and compared the performance of several available docking software and the different algorithms they are based on [12,43,44]. The last step consists in verifying and selecting the most interesting ligands regarding the target. Lots of methods could be applied in order to sort the best molecules: ranking with scoring functions, RMSD comparison with already known ligands, ROC curves to highlight the efficiency of active and inactive compounds separation, etc. Bender and collaborators provide a general and practical guide for the treatment of large-scale docking [45]. We have to notice that some software suites as MOE [46] or the one proposed by Schrödinger (Maëstro [47] and the associated stand-alone tools) offer the possibility to prepare target protein and chemical libraries, then performing docking calculation and analyze the results within the same environment.

### 2.2 Statement of docking data sharing

#### 2.2.1 Current statement

Sharing data is nowadays a great stake to consider in modern science. Numerous concerns have been raised for researches that were nor reproductible, nor replicable, as stated for example by K. Hinsen [48,49]. There is a confusion between the model, the generated data and the software used to generate them, creating a black-box paradigm. It means that computational techniques tend to become simple routine tools, without any need to understand further the implemented algorithms or the scientific backgrounds. One of the solutions is to further develop the sharing of every kind of data, starting from input files and the parameters from the software used to the raw results. That is a statement shared by other groups worldwide, that led to the establishment of the FAIR principles in 2016 to guide the sharing of data in science [15]. It consists in fact of several good practices for data sharing, from the format, the trackability, the reproducibility and the understandability of generated computational data. Destroying the barrier between the results described in a paper and the raw data and providing access to the input files could pave the way to a better comprehension of these computational methodologies by users.

Inspired by those principles, many studies and debates have emerged. Looking to the bioinformatics and cheminformatics fields, much of the focus resolved around the treatment of MD data. Thus, workshops [50], servers for online simulations [51,52] and also storage and listing service of MD trajectories [53,54] have been proposed in the last 5 years. GPCRmd [55] is the most known MD online data repository, containing the MD trajectories of all class of GPCR in which every datum is publicly available.

MD data generation in accordance with the FAIR principles have also been recently proposed. Indeed, Simulation Foundry, proposed in 2020 [56], is an automated workflow on MD trajectories. It highlights the implementation of the FAIR principles within the treatment of the data for each step of a simulation (input files, data generation, output files, analysis).

For docking data, there are some online resources available to assist users in their docking projects. Although their uses are not mandatory, we believe that incorporating those resources for assessing a personalized docking protocol consists in a good practice in this kind of scientific project. We can for example cite the PDBbind database [57], containing several protein-ligand structures with known experimental affinities, but also LIT-PCBA, a curated dataset for virtual screening and machine learning [58] and the DUD-E [59], a dataset comprising active compounds by targets and associated decoys. Furthermore, a new breakthrough is the proposal of entire datasets in ready-to-dock format, following the most classical parameters of physiological environment, as proposed for example by the ZINC database [27]. Obviously, the initial dataset could be downloaded by users who need to prepare it in another way. Building upon this trend, additional datasets, comprising even billions of molecules, are emerging. The most notable one is VirtualFlow [60] which encompasses a workflow for ligand preparation and a ligand dataset comprising 1.4 billion commercially available compounds in ready-to-dock format. The workflow is compatible with lots of docking software and scoring functions, and respects all along the FAIR principles. Both ligand dataset for docking calculations by the user, and entire workflow of the VirtualFlow program, are freely available. In addition, through an open-access GNU GPL license, everyone can contribute to improve this workflow, available on GitHub (http://www.github.com).

However, the sharing of docking results is typically not a common practice, and there are currently no dedicated storage websites designed for depositing and sharing docking data.

#### 2.2.2 Discussion and prospects for a better sharing of docking data

What can be done about the sharing of docking data? Before that, the first question to ask is: what can be defined as “docking data” to share? For example, ligand poses certainly constitute important data to share, but how many of them must be considered? Does it require the structure of the receptor in addition? These are important questions to answer before thinking of sharing. As described in Fig. 1, those kinds of data could be used for analysis applied to projects or reusing or reproducing for methodological development and other modelling techniques. For the first case, only the ranking and the pose of the ligand (maybe the protein structure in some cases) are needed, but for the second kind of reusing, the data origin, generation and results format must be fully transparent.

At first glance, it is evident that certain mandatory data should be systematically provided. This includes the structural information of ligands poses, details about the receptor used and its conformation (one or more). Nonetheless, the ranking according to the scoring is of importance for development of new scoring functions and their possible evaluation on already tested datasets. Consequently, it is crucial to systematically include in the date repository all types of data generated from the docking calculations; this includes the presence of the input files, a detailed description of the various parameters and protocol. Regarding the number of poses, we recommend that at least the top 5 docking poses (ranked based on the score), should be provided, with a special section to highlight the top score pose. The top 5 can be considered for large docking campaign, with a huge number of docked compounds during the virtual screening process in order to save data storage, but a top 10 can be envisioned for small campaign (less than 10k molecules).

Indeed, the results of docking projects are very rarely shared, despite the immense benefit it holds for the scientific community, in order to accelerate drug discovery and create collaborations. Especially nowadays, with the democratization of the online data storage, the rise of the Big Data and the data science, making such results available to the scientific community is of great importance. One could imagine a specialized repository containing the results of large-scale docking, such as ZINC database or others, against one or several members of protein families. The results could be made downloadable by the user, and ranked using several criteria, such as different scoring functions, or experimental data if they exist. Currently, general storage servers are commonly used for this purpose, such as Zenodo (http://www.zenodo.org, accessed on May 5^th^ 2023) or Dryad (http://www.datadryad.org, accessed on May 5^th^ 2023), but docking data remains relatively scarce on those online sharing platforms.

However, the main and easily anticipated problem of this kind of architecture is related to the storage of such big-size data. Indeed, dataset of millions, even billions of compounds take large space in terms of storage, especially when considering several poses for each ligand. This problem has been relevantly discussed by Hospital et al. [61]. Their interesting review focused on the MD data, which are obviously more affected by the size issue due to their inherent nature. Nonetheless, they highlighted two important points to tackle the storage of data: the need of a standard format for the files and their compression processes in order to gain space. They also underlined the need for a long-term sustainability plan in order to safely store the MD data on specific repositories. Such considerations must be kept in mind when applying those principles to the field of docking data sharing.

## 3 Key Points

CADD is now an important field in life science research, owing to the technological progress made during this last decade. Virtual screening, using docking approaches, remains the main methodology for drug discovery processes. Our objective here is twofold: i) to sensitize users of docking methodologies regarding the key aspects for generating relevant docking data, from the preparation of inputs to the verification of results and ii) to highlight the current practices in sharing data of docking calculations and to propose potential future avenues for enhancing data sharing in this field.

We present below some guidelines to follow to generate clear, relevant and reusable data that could be share following the FAIR principles:

• A precise, unambiguous description of the protein structure used, and software employed if applicable (with or without structure minimization, but clearly noted), with date and place.
• If a molecular dynamics approach or other method is used to generate conformers, the set of parameters used for the simulation and also for the structure clustering must be available. It is advisable to have at least the different configurations and if possible, the trajectories.
• A precise description of the origin of the libraries, the protocol and the software used with their versions for the 3D conversion, the refinements used to generate the entire dataset.
• Docking results must be proposed entirely, in order to be reused for any kind of work. This includes a clear listing of algorithm parameters, box size, scoring function, software, the top score pose and top 5 (or 10 depending of the campaign size) ranked poses for each ligand. Receptor's structure must also be provided, with the details of flexibility if applicable.
• In the case of personal protocol that has not been yet verified, its validation must be described, with the dataset used (PDBbind, LIT-PCBA, etc.) and the detail of all the parameters.

All the data generated through this process could then be deposited on a dedicated repository, facilitating access to the data for free among researchers. The question of file format must also be addressed: for example, we suggest that SDF format is the most interesting one for compounds conformations and docking results, but it can be discussed. However, the main difficulty here is identical to that of MD simulations results, the place that raw data can take and their long-term sustainability. Regarding the storage itself, some solutions already exist, such as Zenodo, a storage server granted by the CERN, Dryad or Open Science Framework (OSF, http://www.osf.io, accessed on May 5^th^ 2023). Those storage servers can possibly host docking data and provide an effective solution for researchers who wish to share their data while adhering to the FAIR principles. Actually, only few data from docking calculations are available on those servers, all of them concerning screening of ligand dataset against SARS-CoV2-2019 proteins. There is currently a lack of homogeneity in the format and style of data across different depositories. However, our guidelines can serve as an initial framework for the future deposition of docking data.

As emphasized earlier, the free availability of such data is of crucial importance in today's scientific landscape and could be used for the development of several methodologies, such as deep learning approaches applied to docking and analysis of protein-ligand interactions [62].

We hope that the description provided here can serve as an exemplary model, encouraging researchers to share data from docking calculations. We strongly believe that the entire CADD scientific community could greatly benefit from a more systematic approach to storage and availability of all types of docking results.

## CRediT authorship contribution statement

**Samia Aci-Sèche:** Investigation, Writing – review & editing. **Stéphane Bourg:** Investigation, Writing – review & editing. **Pascal Bonnet:** Writing – review & editing. **Joseph Rebehmed:** Writing – review & editing. **Alexandre G. de Brevern:** Conceptualization, Methodology, Writing – review & editing. **Julien Diharce:** Conceptualization, Methodology, Writing – review & editing, Supervision.

## Declaration of Competing Interest

The authors declare that they have no known competing financial interests or personal relationships that could have appeared to influence the work reported in this paper.
